# Rupture of contralateral mainstem bronchus during uniportal video-assisted thoracoscopy surgery lobectomy and 3 successful cases of repair

**DOI:** 10.1186/s13019-021-01507-w

**Published:** 2021-05-13

**Authors:** Zhilin Luo, Tianhu Wang, Hong Zhang

**Affiliations:** Department of Thoracic Surgery, The Third Affiliated Hospital of Chongqing Medical University, Chongqing, 401120 China

**Keywords:** Uniportal VATS, Bronchial rupture, Repair

## Abstract

**Background:**

Our goal was to discuss the treatment for rupture of contralateral mainstem bronchus during uniportal video-assisted thoracoscopy surgery (uniportal VATS) lobectomy.

**Case presentation:**

We analyzed clinical data of 3 cases of rupture of contralateral mainstem bronchus during uniportal VATS. Surgical repair was performed immediately under an uniportal VATS during operation, as a result, 3 cases of bronchial rupture all were repaired successfully, and we continued to complete lobectomy and systemic lymph node dissection. Reexamination was performed after 1 week, and no fistula was found in trachea and bronchi through a fiberoptic bronchoscopy. The time range for indwelling the chest tube is 6–9 days, and the hospital stay is 8–10 days. No abnormality was observed on chest radiography when the 3 patients returned to the hospital 1 month after the operation for the second reexamination.

**Conclusions:**

Instant surgical repair is recommended to the treatment of bronchial rupture in thoracic surgery. It is safe and feasible to repair bronchial tear with uniportal VATS.

## Background

In thoracic surgery, in order to facilitate the operation, placement of a double-lumen endotracheal tube is needed to ensure the isolation of lungs. During the process, the occurrence of tracheobronchial injuries (TBI) is quite difficult to treat, and sometimes even life-threatening. It has been reported previously in the literature that the incidence of airway injury caused by double-lumen endotracheal intubation was very low, about 0.2% [1]. Airway injury is a rare complication, and sometimes a relatively small bronchial tear associated with that is not easy to be found. The causes include the lack of experienced anesthesiologists, accidental injuries associated with operating of surgeon, anatomical malformations of tracheal bronchi in themselves, repeated intubation, and excessive balloon inflation, etc. In this paper, our goal was to discuss the treatment for rupture of contralateral mainstem bronchus during uniportal VATS lobectomy.

## Case presentation

In this paper, the authors counted the occurrence and treatment of all 3 cases of bronchial rupture in 1075 cases of pulmonary surgery from June 2016 to July 2019 in our hospital (The Third Affiliated Hospital of Chongqing Medical University, Chongqing, China).

All 3 patients are female, and age ranges from 54 to 74 years, and height ranges from 150 cm to 163 cm, and weight ranges from 59 kg to 74 kg. All the 3 patients were diagnosed to have lung occupying lesions by preoperative computed tomography (CT) examination. The pathological diagnosis of percutaneous needle biopsy was confirmed as peripheral lung cancer, of which 2 patients were lung cancer of right lower lobe and 1 patient was lung cancer of right upper lobe. No indicate of distant metastasis was revealed with PET-CT in preoperative examination of 3 patients; no surgical contraindications was indicated in lung function, cardiac function, electrocardiogram, coronary CT angiograph; pulmonary blood vessels, trachea and bronchi can be dissected, which can be seen on chest enhanced CT; and no abnormality was observed in trachea and bronchi through preoperative fiberoptic bronchoscopy. In terms of comorbidities, there was 1 patient complicated with rheumatoid arthritis, with receiving long-term immunosuppressive agents, 1 patient with coronary heart disease, and the other one with no comorbidity. With preoperative discussions by multidisciplinary team (MDT), all 3 patients were identified to have surgical indications and no surgical contraindications.

The anesthesiologists for 3 cases are experienced of more than 20 years. General anesthesia was induced by Sufentanil, Propofol, Rocuronium Bromide, and Etomidate. Before intubation, on comprehensive consideration of above-mentioned height and weight of patients and measurements of the bronchial diameter on the preoperative chest CT imaging, double-lumen bronchial tube No.35 was prepared for 2 patients and No.37 for the other one. Once the tip of the tube had passed through the cords, the guidewire was removed, and then the tube was rotated 90 degrees counterclockwise and went forward. The intubation process was smooth and no resistance was encountered during placement. Finally, the position of the tube was confirmed by the fiberoptic bronchoscopy. After the whole intubation, patient took the left lateral position. After changing body position, the position of the tube was confirmed again, with no bleeding in the airway, at this point, the intubation was satisfactory.

**Fig. 1:**
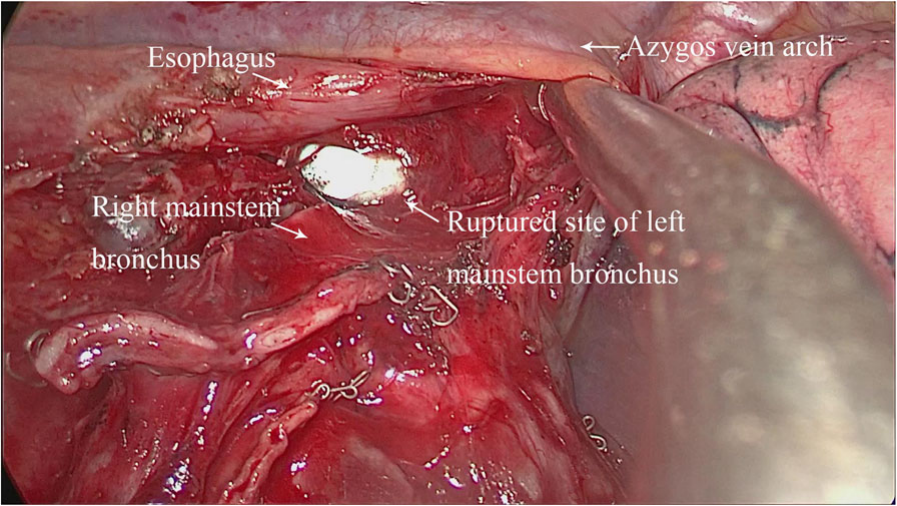
Ruptured site of left mainstem bronchus before the repair

Because of the preoperative pathological diagnosis to identify clearly lung cancer, all 3 patients underwent right lower lobectomy and systemic lymph node dissection with uniportal VATS. The operative position was taken in the left lateral position, and the incisions, with a length of approximately 3 cm, were located at the fourth intercostal space on the right side, anterior axillary line or mid-axillary line. Lymph node dissection covered station 2, 4, 7, 8, 9, 10, and 11. Station 7 was resected as following: after the right lung collapsed, clamp the gauze pad with the oval clamp to press the lung towards anterior mediastinum in order to fully reveal the posterior mediastinal structure. The posterior mediastinal pleura was then opened with an ultrasonic knife to reveal the subcarinal structure for the dissection of station 7. In this paper, it was founded that there were a longitudinal rupture of the left mainstem bronchus, with a length of 3 cm, and balloon of the tracheal intubation inside to 3 patients after the posterior mediastinal pleura opening. Patients had obvious emphysema under the posterior mediastinum, but the balloon of tracheal intubation was intact (Fig. 1).

**Fig. 2:**
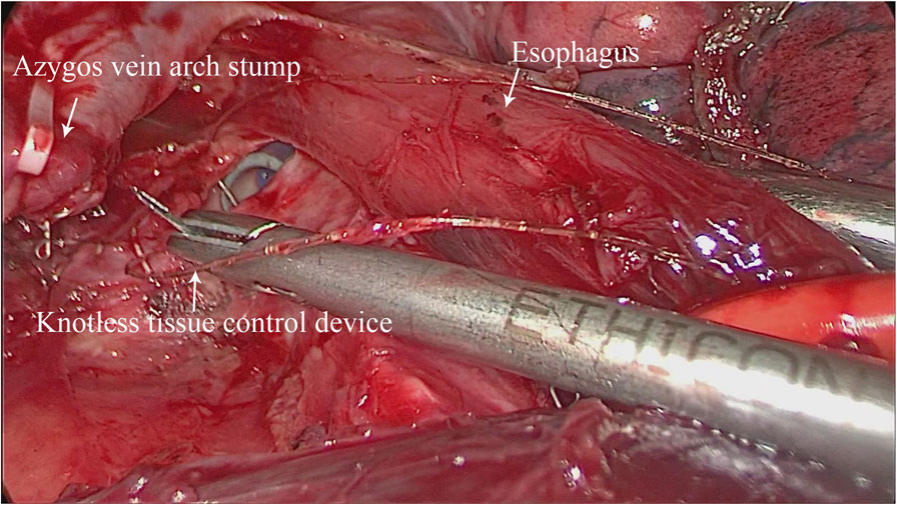
During the process of the repair

**Fig. 3:**
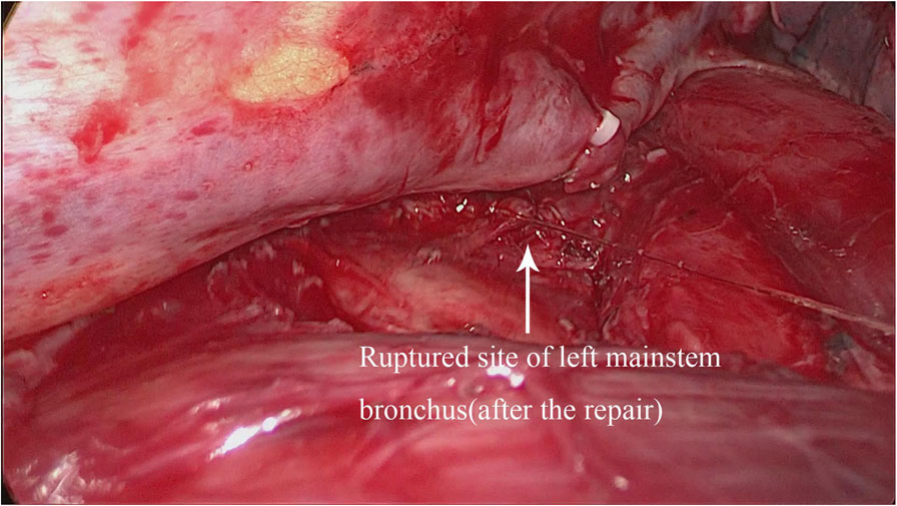
After the repair. There is no air leak identified through water testing

Repair process: Continued uniportal VATS left mainstem bronchial repair under the original single-port incision. The subcarinal structure was fully dissociated to reveal the left and right mainstem bronchus. The azygos vein arch was dissected by using a linear stapler, and then the esophagus near the carina was fully dissociated and pulled towards anterior mediastinum. After fully revealing the ruptured site of left mainstem bronchus, it was sutured with knotless 3–0 stratefix spiral continuously (Fig. 2, Fig. 3). The following points should be noted while suturing: 1. Suture from the distal end of the ruptured site to the proximal end; 2. Choose knotless 3–0 stratefix spiral with a radian of 1/2C to shorten the operation time as much as possible; 3.During the suturing process, pay extra attention to the suction of blood and exudation on operated site of chest, so as not to flow into the contralateral bronchus through the bronchial rupture; 4.While suturing, closely cooperate with the anesthesiologist, that is, when insert the needle, the tracheal tube should be retracted by 0.5 cm to leave space for suturing; after removing the needle, the tracheal tube is pushed forward towards the distal end of the endotracheal tube to completely block the bronchial rupture. This is to not only satisfy the requirement for suture but also ensure the ventilation of the contralateral lung and the collapse of lung on the operated side. After completing the suturing, no air leak was observed during water testing and lung inflation. After completing the repair of the left mainstem bronchus, right lower lobectomy and lymph node dissection with uniportal VATS were continued. By the end of the operation, the operation time ranges from 203 to 256 min of the 3 patients, and the blood loss ranged from 110 to 175 ml.

**Fig. 4:**
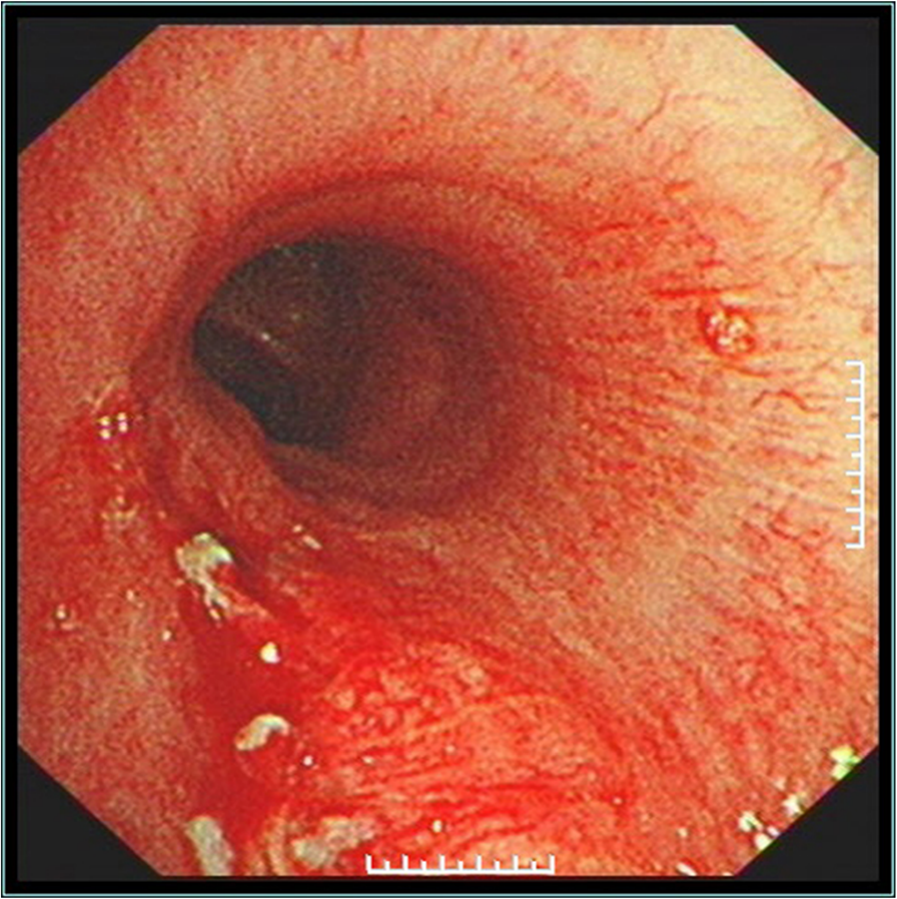
Left mainstem bronchus under fiberoptic bronchoscopy 1 week after operation

**Fig. 5:**
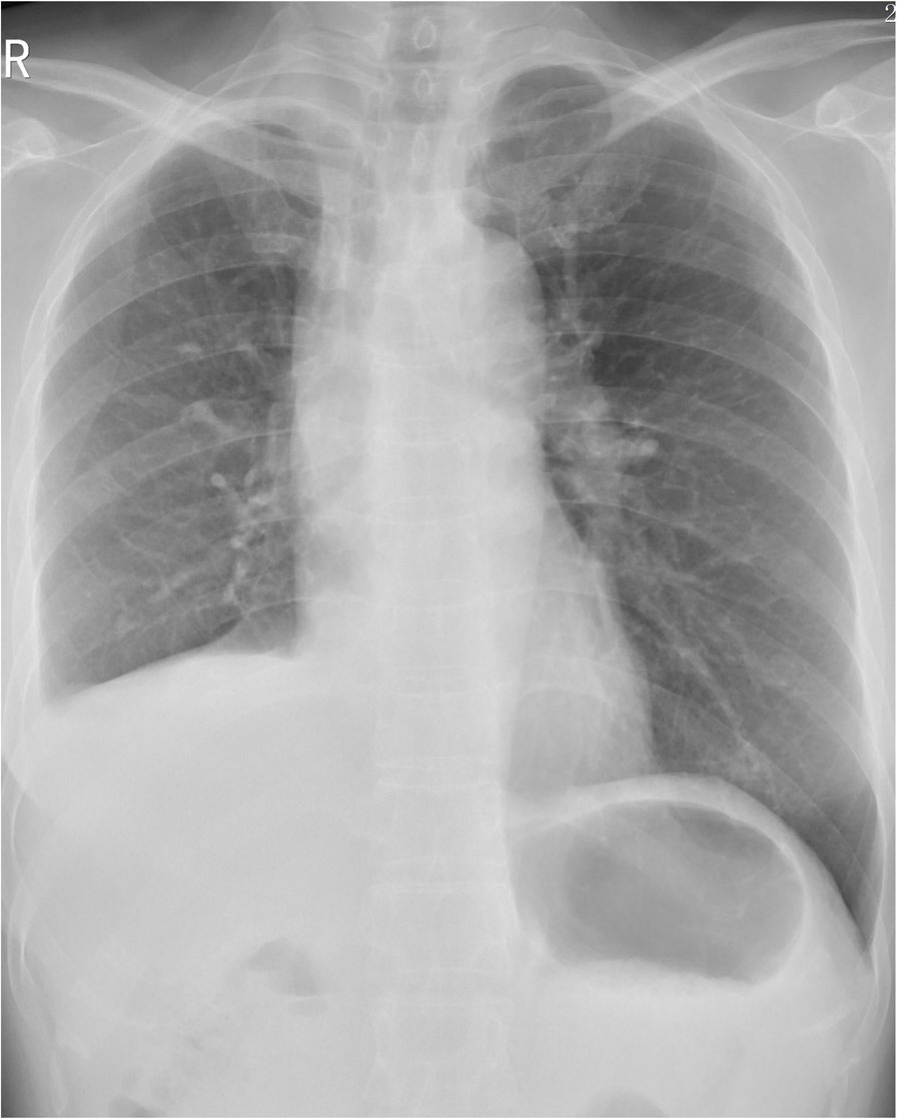
Reexamine with chest radiography half a month after discharge, no abnormality was observed

The vital signs of patients were stable during the operation, on the day, patients were transferred to Intensive Care Unit (ICU) for monitoring and treatment after operation, and returned to the general ward the next day. One week after the operation, congestion and edema were found to left bronchial mucosa, but no fistula was seen (Fig. 4). The time range for indwelling the chest tube is 6–9 days, and the hospital stay is 8–10 days. Reexamine with chest radiography half a month after discharge, No abnormality was observed (Fig. 5).

## Discussion

Double-lumen endotracheal intubation, which is essential in most of current thoracic surgery, can isolate lungs with a result that the surgical side lung collapses in order to facilitate operating. However, before intubation, the anesthesiologist should carefully observe the results of preoperative bronchoscopy, and identify anomalous airway anatomy or airway stenosis on the chest CT. It is noted to select tracheal tube with an appropriate diameter, and move gently while intubating to reduce the tracheal injury caused by violent intubation. After intubating, try to inflate the bronchial cuff with the lowest volume to obtain lung isolation. At the same time, if necessary, the bronchial cuff pressure should be monitored for the maintenance of < 35-40cmH_2_O [2].

With completing the intubation of a double lumen endotracheal tube, there are many causes for a tracheobronchial rupture, including iatrogenic factors such as accidental injuries from anesthesiologists, surgeons, and anatomical malformations of tracheal bronchi in themselves, which should be avoided during clinical treatment. The 3 cases of intraoperative left mainstem bronchial rupture occurred in this paper are considered to be related to the repeated position adjustment of the tracheal intubation due to poor intraoperative lung collapse, and factors like the excessive balloon pressure are not excluded. A small rupture associated with TBI may not be founded during operation timely, however, if there is a large amount of subcutaneous emphysema, mediastinal emphysema, decreased breath sounds, difficulty breathing, etc., examination with chest CT, fiberoptic bronchoscopy or the related should be performed in time for a confirmation. Certainly, Instant surgical repair is recommended to the treatment of TBI identified in operation.

VATS, especially uniportal VATS, has been confirmed as effective to achieve radical resection of pulmonary carcinoma as two-portal thoracoscopy surgery and open surgery, but even with smaller wound [3, 4]. If a bronchial rupture occurs with uniportal VATS, the preferred attempt is still to repair under a single port. However, an appropriate needle should be selected when repair a bronchial tear with an uniportal VATS, due to the narrow operation space, and the knotless suture should be selected as much as possible to save the operation time. At the same time, an anesthesiologist’s close cooperation is needed to ensure the collapse of the lung on surgical side and good ventilation of the contralateral lung. If it is difficult for the operator to operate with an uniportal VATS due to surgical experience, skills, etc., additional operating port or transfer to thoracotomy can be recommended, because the timely successful repair of bronchial tear is of great importance.

For patients with tracheobronchial rupture repair, we recommend: 1. If the patient has nutritional risks, adequate nutritional support is needed; 2. Chest radiograph should be reexamined in time for the situation of the lung recruitment; 3. Postoperative fiberoptic bronchoscopy should be performed in time to show condition of airway after suturing for identification of tear; 4. Patients should be asked to strengthen cough to facilitate sputum drainage, if necessary, sputum suction can be performed under a fiberoptic bronchoscopy to ensure airway patency, and antibiotics should be adjusted timely according to results of sputum culture, parameters of blood routine, procalcitonin and other indicators; 5. For patients with diabetes, plasma glucose should be controlled, if necessary, please consult the department of endocrinology and consultation for assistance.

## Conclusions

1. Instant surgical repair is recommended to the treatment of bronchial rupture in thoracic surgery. 2. It is safe and feasible to repair bronchial tear with uniportal VATS.

## Data Availability

Please contact the corresponding author to request this information.

## Abbreviations

VATS: Video-assisted thoracoscopy surgery
TBI: Tracheobronchial injuries
ICU: Intensive Care Unit
MDT: Multidisciplinary team
CT: Computed tomography
